# Mapping a differentiation architecture for hard tissue mineralization with large-scale single-cell atlases

**DOI:** 10.1371/journal.pcbi.1014788

**Published:** 2026-09-21

**Authors:** Litian Han, Yiqian Yu, Mengge Feng, Zishu Lin, Yulan Wang, Ting Xia, Qihang Fan, Huan Liu, Yan Wei, Yufeng Zhang

**Affiliations:** 1 Key Laboratory of Oral & Maxillofacial Reconstruction and Regeneration, Key Laboratory of Oral Biomedicine, Ministry of Education, Hubei Key Laboratory of Stomatology, School & Hospital of Stomatology, Wuhan University, Wuhan, China; 2 Medical Research Institute, School of Medicine, Wuhan University, Wuhan, China; 3 Taikang Center for Life and Medical Sciences, Wuhan University, Wuhan, China; University of Alabama at Birmingham, UNITED STATES OF AMERICA

## Abstract

Mineralization is a critical process in the formation of hard tissues such as bones and teeth, yet the cellular mechanisms underlying this process remain incompletely understood. To address this, we constructed a comprehensive single-cell atlas of tooth development, integrating data from 261,929 cells across 15 projects and encompassing both odontogenesis and amelogenesis. We developed a novel algorithm, TrajDTW, to identify genes with concordant trajectory dynamics across these large-scale datasets, allowing us to detect robust developmental signals. To define a shared mineralization trajectory architecture, we then integrated our tooth atlas with a previously constructed bone atlas. Applying TrajDTW to this combined resource revealed common molecular pathways governing the formation of distinct hard tissues, including bone, enamel, and dentin. Furthermore, cross-species analysis between human and mouse data uncovered cross-species conserved mesenchymal/odontoblast programs. This study provides an unprecedented resource for developmental biology and defines a molecular framework for mineralization that is shared across tissues and species.

## Author summary

The fundamental genetic rules governing hard tissue formation have long been elusive. In this study, we define a shared mineralization trajectory architecture by creating the most comprehensive single-cell atlas of tooth development to date. We developed a novel algorithm to identify trajectory-concordant gene programs across large-scale datasets and applied it to integrate our tooth atlas with an existing bone atlas. This integrated, cross-species analysis pinpointed the common molecular machinery directing hard-tissue formation. Our work provides an unprecedented resource and a powerful analytical framework, laying the foundation for new regenerative strategies aimed at treating skeletal and dental disorders.

## Introduction

Mineralization is a fundamental biological process that underpins the development of hard tissues, such as bones and teeth, through the deposition of inorganic minerals, primarily hydroxyapatite, onto an organic matrix [1–3]. In bones, this process, known as osteogenesis, provides structural support and facilitates movement, while in teeth, odontogenesis leads to dentin formation, and amelogenesis results in the production of enamel, the hardest tissue in the human body [4–6]. Despite their distinct anatomical and functional roles, bone and tooth mineralization share notable similarities, including the involvement of calcium phosphate crystals and extracellular matrix proteins, suggesting potentially shared cellular and molecular mechanisms [2,6–8]. Understanding these shared pathways could illuminate developmental biology and inform regenerative therapies for skeletal and dental disorders [9,10].

The emergence of single-cell RNA sequencing (scRNA-seq) has revolutionized developmental biology by enabling high-resolution profiling of gene expression at the individual cell level [11,12]. This technology has facilitated the creation of comprehensive cellular atlases for various tissues, exemplified by initiatives such as the Human Cell Atlas, which systematically map cellular diversity and developmental trajectories [13]. However, despite the critical roles of teeth in mastication, speech, and overall health, a comprehensive single-cell atlas encompassing the complete spectrum of tooth development—including both odontogenesis and amelogenesis—has been notably absent from the literature. While previous studies [14,15] mapping human and mouse dental tissues have provided valuable insights, they remain limited in scale and scope, highlighting the need for a more comprehensive and integrative approach.

Trajectory inference methods in scRNA-seq analysis enable researchers to reconstruct developmental pathways and identify key regulatory genes that drive cellular differentiation [16]. However, a significant computational challenge in leveraging large-scale single-cell datasets lies in identifying genes with concordant trajectory dynamics across diverse samples, species, or experimental conditions, primarily due to technical noise and inherent biological variability [17–19]. Existing algorithms [20,21] for trajectory analysis often struggle to effectively handle the complexity of large-scale datasets, underscoring the critical need for novel computational tools specifically designed to detect robust developmental signals. To date, no algorithm has been specifically developed to identify trajectory-concordant genes across extensive single-cell datasets from multiple independent projects—a significant gap that our study aims to address.

In this study, we constructed a filtered core single-cell atlas of tooth development comprising 261,929 cells from 31 samples across 15 projects, capturing the dynamic processes of both odontogenesis and amelogenesis. We further assembled an extended atlas comprising 391,327 cells from 59 samples across 21 projects to support broader cross-dataset and cross-species analyses. We developed a novel computational algorithm to identify genes with concordant trajectory dynamics across these datasets, enabling the detection of the most robust signals in developmental processes. Crucially, our tool’s utility extends beyond conservation in differentiation, proving applicable to other biological phenomena, such as the cell cycle. By combining our comprehensive tooth atlas with an existing bone atlas, we identified a shared mineralization trajectory architecture that elucidates common molecular mechanisms underlying hard tissue formation. In a cross-species analysis restricted to mesenchymal/odontoblast-related populations, we identified selected human–mouse orthologs with conserved pseudotemporal dynamics, offering new insights into the cross-species concordant mineralization patterns of hard tissue development. Our work provides a detailed molecular map of tooth development at single-cell resolution, bridges the existing gap between bone and tooth mineralization studies, and reveals both shared and species-specific features of these critical biological processes.

## Results

### Construction of a comprehensive single-cell atlas of tooth development

We initially collected a core set of healthy tooth-related datasets comprising 339,489 cells from 35 samples across 16 projects. Following quality control, including removal of doublets/non-singlet cells and exclusion of one dataset that did not meet our quality criteria, the filtered core atlas used for primary downstream analyses comprised 261,929 cells from 31 samples across 15 projects. These datasets span mouse development from embryonic stages to adulthood, covering tissues from embryonic dental germs to dental pulp (Figs 1A and S1). We established a standardized processing pipeline for atlas construction and, after benchmarking multiple integration methods, selected scANVI as the optimal approach for dataset integration [22,23] (Methods, S2A and S3A–S3H Figs). All projects were successfully integrated, and the final atlas comprised eight major cell clusters: endothelium, epithelium, immune, mesenchyme, muscle, neuron, perivascular, and red blood cells (Figs 1B, S4A and S4B). The complete processing pipeline is documented at https://scatlas.readthedocs.io/en/latest, and an interactive version of the atlas is available at https://zyflab.shinyapps.io/tooth.

**Fig 1:**
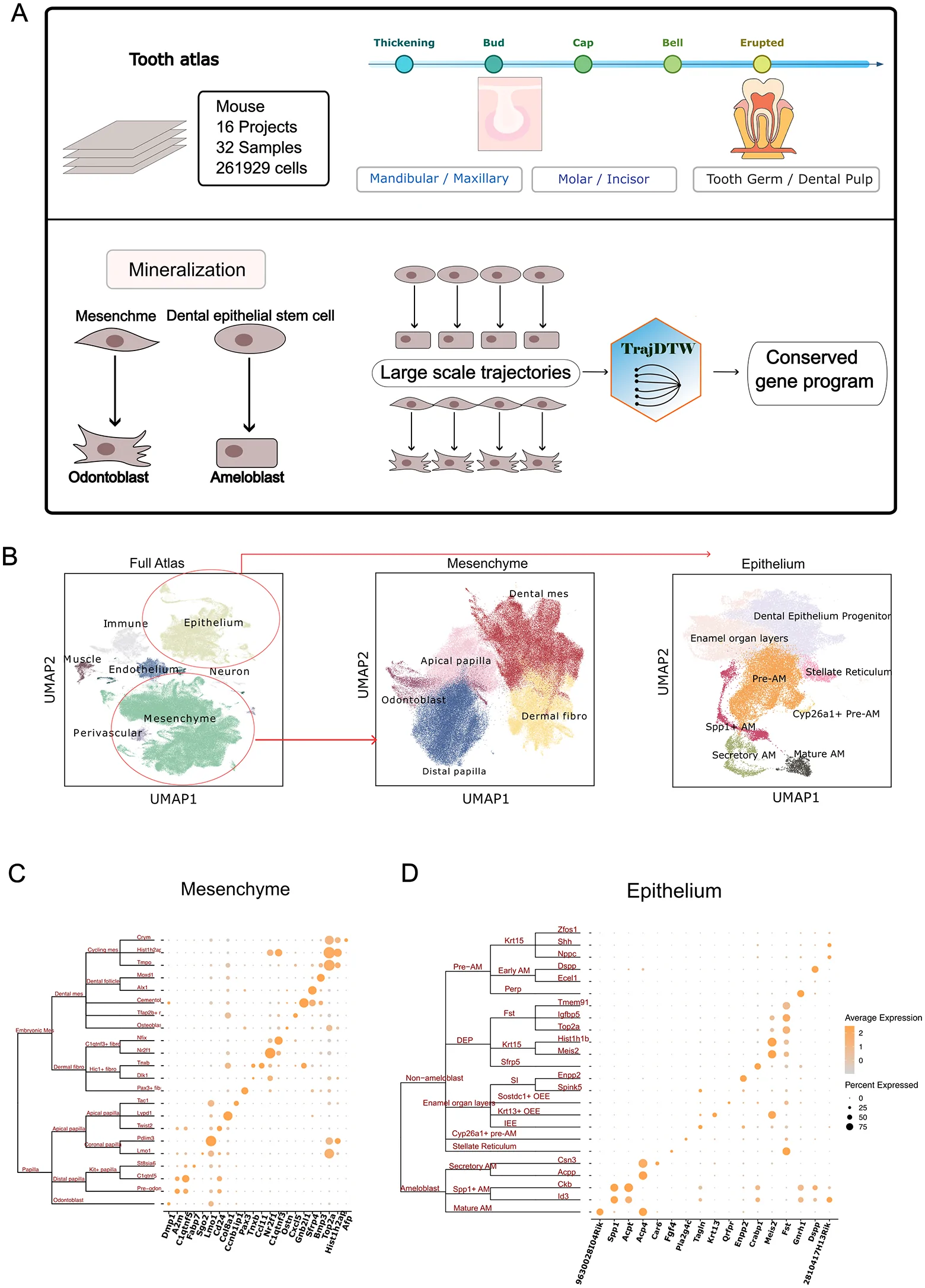
Construction of a comprehensive single-cell atlas of tooth development. (A) Atlas construction overview. The top panel illustrates the datasets collected across various developmental stages. The bottom panel outlines the analytical strategy. Our analysis focuses on the mineralization process, for which we developed TrajDTW, a novel software tool for integrating large-scale trajectory data to identify trajectory-concordant gene programs driving differentiation. (B) Dimensionality reduction of the integrated atlas. UMAP (Uniform Manifold Approximation and Projection) plots visualize the complete atlas, as well as the segregated Mesenchyme and Epithelium compartments. (C, D) Hierarchical annotation of Mesenchyme and Epithelium. The hierarchical clustering trees on the left depict a four-level annotation of cell subtypes within the (C) Mesenchyme and (D) Epithelium. The corresponding dot plots on the right display the expression patterns of canonical marker genes used to identify each cluster.

Mesenchyme and epithelium constitute the largest cell proportions within our atlas (S4A and S4B Fig). Given our focus on mineralization, we selected these two populations for deeper analysis. We constructed our annotation system using a multi-level clustering strategy, and using CellHint [24] to prove that our cell labels were harmonized across studies and maintained consistent annotations (S4D Fig). Our reference atlas provides comprehensive insights into dental cell taxonomy, including well-characterized cell types (Fig 1C). Within the mesenchyme, we identified major cell states such as dental mesenchyme, dermal fibroblast, dental follicle, apical papilla, dental papilla, and odontoblast, which align with most published literature [15,25,26]. We also pinpointed more subtle cell states, like *C1qtnf3* + fibroblasts [27] and *Kit*+ papilla cells [28], previously reported in only a few studies.

For the epithelial population, our analysis achieved very high resolution in cell state differentiation (Fig 1D). We defined outer enamel epithelium using the marker *Slco4a1*, inner enamel epithelium with *Sfrp5*, and stratum intermedium with *Jph4*. Significantly, our atlas allows for the distinction of ameloblasts into discrete developmental states: pre-ameloblasts, marked by *Col22a1*; early ameloblasts, identified by *Dspp*; secretory ameloblasts, indicated by *Enam*; and mature ameloblasts, defined by *Odam* (Fig 1D). This evidence confirms the broad coverage and high resolution of our atlas, advancing any tooth atlas [15,29] published to date.

We elucidated a significant distinction between embryonic and adult mesenchymal tissues, which can be respectively distinguished by the specific markers *Prrx1* (embryonic) and *Msx2* (postnatal) **(**S5A Fig). Interestingly, despite this overarching difference, both the apical papilla and dental follicle exhibit potentially shared cellular and molecular mechanisms across these two life stages, as defined by their respective markers, *Smoc2* and *Bmp3* [25]. Our comparative analysis further delineated the functional divergence between the embryonic and adult tissues (S4 Table). In the dental follicle, upregulated genes in the embryonic stage were predominantly enriched in Gene Ontology terms for neuron development (S5E Fig). This implicates a strong link between the nervous system and the regulatory networks governing embryonic mineralization (S5E Fig). The adult follicle, by contrast, was enriched in pathways related to the collagen-containing extracellular matrix, suggesting its mature function is primarily focused on the structural maintenance and remodeling of the periodontium (S5E Fig). In the apical papilla, we observed that *Igfbp2* was significantly upregulated in the embryonic tissue (S5F Fig). As a direct target of the transcription factor *Runx2*, the dysregulation of *Igfbp2* has been previously linked to dental abnormalities, highlighting the importance of this finding [30]. Meanwhile, the adult papilla was characterized by an enrichment of genes associated with the collagenous extracellular matrix, including *Lum* and *Col9a2*, pointing towards a functional role in preserving the homeostatic balance and structural framework of the adult apical pulp (S5G and S5I Figs).

### Construction of high resolution odontoblast and ameloblast differentiation pathway

We reconstructed odontoblast and ameloblast differentiation pathways to address two major challenges in developmental biology: the lack of continuous trajectories in previous atlases and an incomplete understanding of how factors like age affect differentiation. Our comprehensive atlas provides the scale needed to construct these detailed pathways, overcoming prior limitations.

We have previously established that embryonic and adult mesenchymal populations occupy distinct cellular states. To elucidate their differentiation dynamics, we performed trajectory analysis, which demonstrated that progenitor-like cells from embryonic dental mesenchyme and adult apical pulp embark on divergent initial paths before converging to a common terminal odontoblast phenotype (Fig 2A and 2B). We next used a common pseudotime coordinate to compare progression from embryonic dental mesenchyme and adult apical pulp toward an odontoblast state (Fig 2A–2C). We therefore fitted condition-specific smooth expression curves and tested for embryo–adult differences in pseudotime-dependent expression. Among 14,209 tested genes, 14,183 yielded valid tests and 1,215 genes (8.6%) met the FDR < 0.05 criterion. The significant genes were organized into eight dynamic programs, revealing condition-specific differences in developmental and signaling processes (Figs 2G and S8A). Notably, the early embryonic differentiation program was significantly enriched for genes governing cell fate commitment (e.g., *Prrx1*, *Wnt16*, *Wnt2*) (Fig 2G). In contrast, the corresponding adult program was characterized by an enrichment of factors involved in the “Regulation of Wnt signaling pathway,” including *Fgfr2* and *Wnt5a*, indicating disparate regulatory mechanisms (Fig 2G). Furthermore, the terminal cell states exhibited molecular heterogeneity: embryonic-derived odontoblasts expressed osteogenic-associated genes such as *Mef2c*, *Col2a1*, and *Alpl*, while their adult counterparts were defined by markers like *Phex*, *Bglap3*, and *Smpd3* (Fig 2G). A focused examination of signaling molecules revealed that while some factors (*Notch1*, *Notch2*, *Fgf10*) displayed concordant trajectory dynamics, others were lineage-restricted (Fig 2I). Specifically, *Bmp4* and *Fgf3* expression was confined to the embryonic trajectory, whereas *Notum*, *Wnt5a*, and *Fgf9* were exclusively active in the adult trajectory (Fig 2I). Collectively, these results delineate a profound heterogeneity in both the developmental progression and the underlying signaling networks of embryonic and adult odontogenesis.

**Fig 2:**
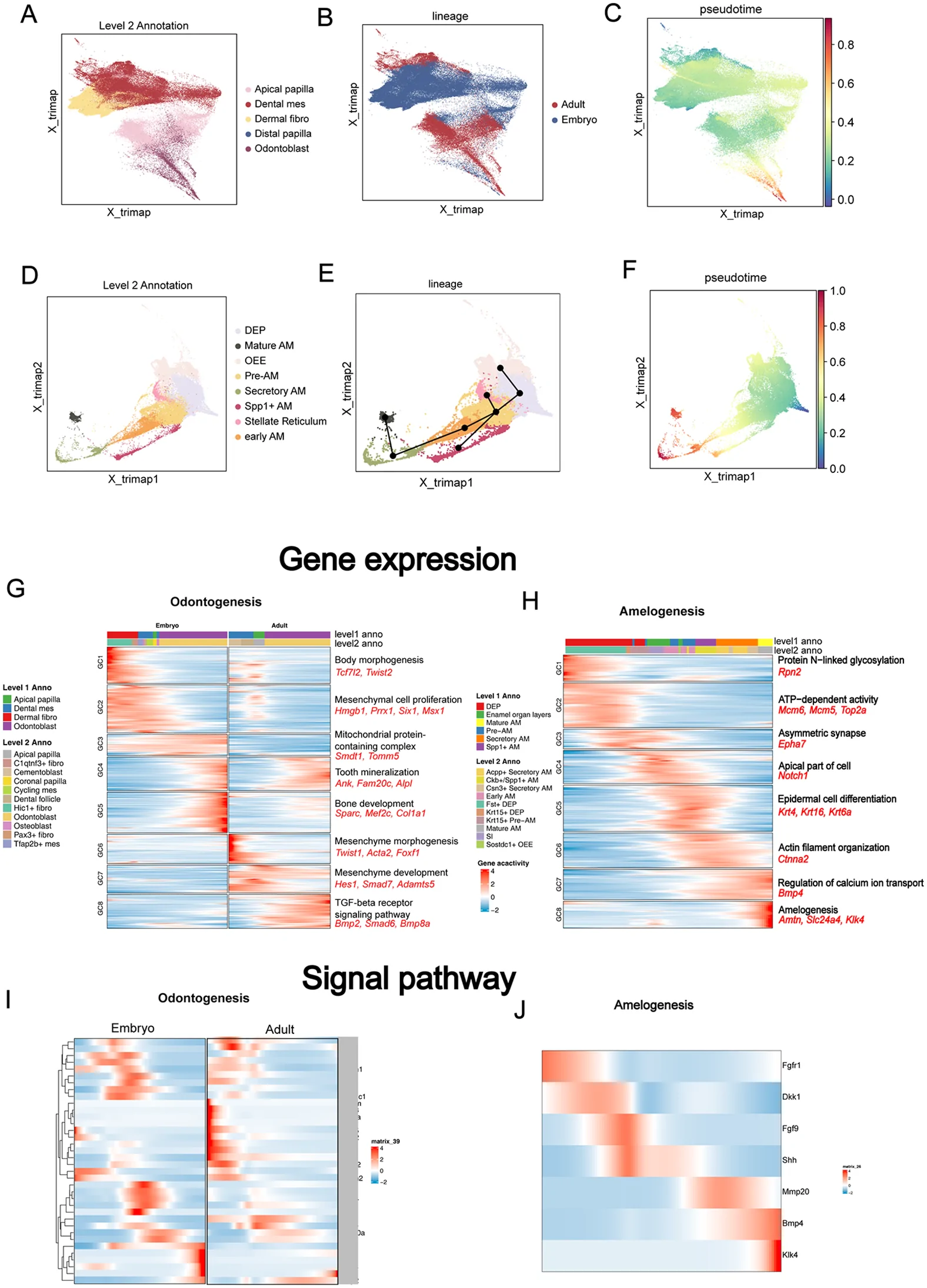
Construction of high resolution odontoblast and ameloblast differentiation pathway. (A, D) Trimap dimension reduction plots illustrating the developmental continuum of odontogenesis (A) and amelogenesis (D). (B, E) Corresponding Trimap plots showing the inferred developmental lineages for odontogenesis (B) and amelogenesis (E). (C, F) Pseudotemporal ordering of cells along the odontogenesis (C) and amelogenesis (F) trajectories. (G, H) Heatmaps of dynamic gene expression changes along the pseudotime axes for odontogenesis (G) and amelogenesis (H). For panel G, condition-dependent genes were identified using tradeSeq::conditionTest applied to condition-specific smoothers. For panel H, pseudotime-associated genes were identified using tradeSeq::associationTest. P values were adjusted separately for each analysis using the Benjamini–Hochberg method (FDR < 0.05). Heatmaps are annotated with sample information (top) and Gene Ontology (GO) enrichment terms (right). The odontogenesis heatmap (G) is separated by embryonic and adult samples. (I, J) Heatmaps depicting dynamic changes in signaling pathway activity over pseudotime for odontogenesis (I) and amelogenesis (J).

In parallel, we delineated the differentiation trajectory of the principal epithelial lineage. The high resolution of our atlas allowed us to capture the entire continuum of ameloblast differentiation, resolving a clear progression: from dental epithelial progenitor-like cells through enamel organ layers, pre-ameloblasts, early ameloblasts, secretory ameloblasts, and culminating in mature ameloblasts (Figs 2D, 2E and S6E). Pseudotemporal analysis of this path also revealed distinct transcriptional phases. The trajectory initiated with an enrichment of genes associated with ATP-dependent cell cycle activity (*Mcm6*, *Mcm5*, *Top2a*) and cell-cell signaling (*Epha7*), indicating rapid proliferation and communication (Fig 2H). The mid-point was characterized by genes driving epidermal cell differentiation, reflecting lineage commitment. Finally, the late differentiation stage was dominated by genes crucial for amelogenesis (*Amtn*, *Slc24a4*, *Klk4*) and regulation of calcium ion transport (*Bmp4*), marking the onset of specialized mineralization functions (Fig 2H). This process was orchestrated by a dynamic signaling cascade, starting with *Fgfr1* and *Dkk1* at the early stage, transitioning to *Fgf9*, *Shh*, and *Mmp20* at the middle stage, and concluding with *Mmp20*, *Bmp4*, and *Klk4* during terminal maturation (Fig 2J).

### TrajDTW identifies trajectory-concordant genes across large-scale datasets

Single-cell datasets are inherently susceptible to technical and biological biases. These confounding factors, stemming from experimental variables such as sampling methods and intrinsic cellular heterogeneity, complicate the robust identification of genes that genuinely reflect underlying biological processes. [22,31]. To address this challenge, we have developed a novel algorithm based on Dynamic Time Warping (DTW) to identify trajectory-concordant gene sets associated with specific biological processes, leveraging the extensive biological replicates within our large-scale atlas. While DTW is conventionally applied to the alignment of paired cellular trajectories [32,33], we have repurposed this framework to specifically detect genes with concordant trajectory dynamics across large-scale developmental trajectories (Fig 3A).

**Fig 3:**
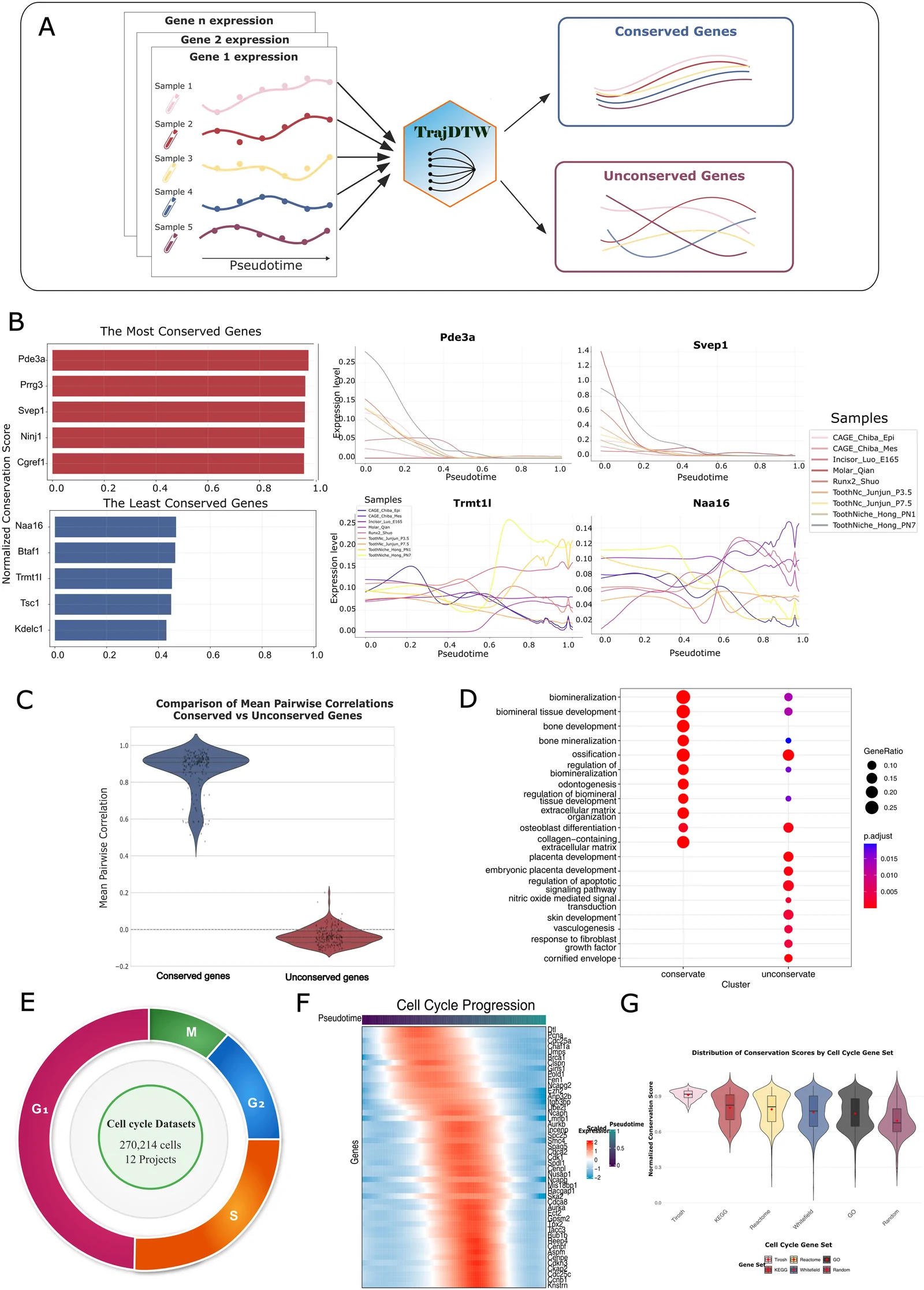
TrajDTW identifies trajectory-concordant genes across large-scale datasets. (A) Schematic overview of the TrajDTW method. (B) Identification of genes with high and low trajectory concordance scores. The bar plot (left) ranks genes by their TrajDTW-derived trajectory concordance score. Pseudotemporal expression plots (right) show representative high-concordance genes with reproducible trajectory dynamics and low-concordance genes with variable trajectory patterns. (C) Pearson correlation analysis of expression profiles for high- and low-concordance genes identified by TrajDTW. (D) Gene Ontology (GO) enrichment analysis of high- and low-concordance gene sets. (E) UMAP visualization of the cell cycle dataset. (F) Heatmap displaying the pseudotemporal expression of trajectory-concordant genes across the cell cycle trajectory. (G) Violin plots comparing the trajectory concordance scores of previously published cell cycle gene sets.

To validate the capability of TrajDTW to detect consistent expression patterns, we applied the algorithm to our odontoblast dataset, which comprises nine distinct trajectories characterized in the preceding section. As hypothesized, trajectories that received high trajectory concordance scores from TrajDTW demonstrated highly consistent expression patterns, whereas those with low scores exhibited substantial variability (Fig 3B). To further substantiate these findings, we performed a quantitative analysis by calculating pairwise Pearson correlation coefficients (Fig 3C). This revealed that genes with higher trajectory concordance scores also had significantly stronger correlation coefficients, thereby confirming the validity and robustness of our algorithmic approach (Fig 3C).

We further evaluated the biological significance of the TrajDTW trajectory concordance score by integrating it with our previous TradeSeq results. Notably, within any given expression pattern identified by TradeSeq, genes with higher trajectory concordance scores were significantly enriched for key differentiation-related Gene Ontology (GO) terms, such as ‘odontogenesis’ and ‘biomineral tissue development’ (Fig 3D). This finding indicates that our trajectory concordance analysis effectively isolates the most biologically meaningful genes from within broader expression clusters. In a parallel analysis, we combined the TrajDTW scores with a differential expression analysis comparing odontoblasts to progenitor mesenchymal cells (S11A Fig). Similarly, genes with high trajectory concordance scores were again strongly enriched for the same biologically relevant GO terms (S11A Fig). Collectively, these results validate that the genes identified as highly trajectory-concordant by TrajDTW are functionally critical to the developmental process under study.

To directly compare TrajDTW with alternative approaches, we benchmarked it against aligned Pearson correlation, coefficient-of-variation similarity, and Genes2Genes using controlled synthetic trajectories. TrAGEDy was not included because its primary output is an alignment between cell-state trajectories rather than a per-gene trajectory-consistency score and therefore does not provide a directly comparable gene-ranking output [32]. Under low noise, TrajDTW and Genes2Genes both achieved near-perfect average precision, whereas CV similarity remained close to the prevalence baseline. At intermediate noise levels, TrajDTW achieved the highest average precision: at σ = 3.0, average precision was 0.815 for TrajDTW, 0.649 for Pearson correlation, and 0.493 for Genes2Genes. At σ = 6.0, however, all methods showed substantially reduced discrimination, indicating the limit of recoverable trajectory signal under the strongest tested noise condition.

Pearson correlation performed similarly or better when trajectories were nearly aligned, but TrajDTW retained higher average precision as temporal displacement increased. At Δ = 0.10, 0.20, and 0.30, mean average precision was 0.862, 0.654, and 0.398 for TrajDTW, compared with 0.750, 0.371, and 0.243 for Pearson correlation. TrajDTW also retained the highest mean average precision at the largest matched warp area across all four nonlinear transformation families. These results indicate that TrajDTW is particularly advantageous under moderate-to-large temporal displacement and nonlinear pseudotime distortion, rather than being uniformly superior under all benchmark conditions (S10D–S10F Fig). In the largest runtime workload(480 genes, four replicates per condition, and 16 cross-condition replicate pairs), median scoring wall time was 37.34 s for TrajDTW and 1,363.76 s for Genes2Genes, a 36.5-fold difference under this benchmark configuration (S10G Fig).

To enhance the validation of our algorithm’s capability to identify functionally coherent gene sets, we conducted an analysis of cellular proliferation datasets. A comprehensive cell cycle atlas was assembled from 270,214 cells originating from 12 distinct research projects. (Fig 3E and S5 Table) The well-defined characteristics of the cell cycle provide an exemplary model for assessing the methodological accuracy of our approach. Following the estimation of the cell cycle phase for each cell, we employed TrajDTW to identify trajectory-concordant genes associated with the cell cycle. (Figs 3F and S11C) A Gene Ontology (GO) enrichment analysis was subsequently performed. The results indicated that genes with high trajectory concordance scores, as determined by TrajDTW, were significantly enriched for cell cycle-related terms in comparison to genes with low trajectory concordance scores (S11D Fig). To further substantiate these findings, we compared our results with established, experimentally validated cell cycle gene datasets, including those from Tirosh et al. [34], KEGG, Reactome, and Whitfield et al. [35] We observed that genes within these reference datasets exhibited significantly higher trajectory concordance scores than randomly selected gene sets (Fig 3G and S6 Table). Notably, the Tirosh dataset demonstrated a mean trajectory concordance score of 0.9, signifying a strong correlation with the gene sets identified by our algorithm (Fig 3G). For secondary validation, we utilized an independent dataset focused on cell cycle activation. Within this dataset, TrajDTW-prioritized high-concordance genes consistently demonstrated expression dynamics that mirrored the established cell cycle trajectory (S11E Fig). Conversely, genes with low trajectory concordance scores displayed divergent or stochastic expression patterns (S11E Fig). These results collectively demonstrate the efficacy of TrajDTW in identifying trajectory-concordant genes across large-scale trajectory data, thereby revealing highly co-regulated and shared patterns during specific biological processes.

### TrajDTW identifies the shared mineralization trajectory architecture across hard tissue

Mineralization is a highly conserved biological process, and the key cell types responsible—osteoblasts, odontoblasts, and ameloblasts—share fundamental developmental pathways. We therefore hypothesized the existence of a “shared mineralization trajectory architecture,” representing the core molecular programs that govern hard tissue formation. To identify this architecture, we utilized our TrajDTW algorithm on a comprehensive cellular atlas (Fig 4A). This atlas was constructed by integrating our novel tooth development atlas with our previously published bone atlas [36], thereby encompassing the primary mineralizing tissues in the mouse. To mitigate potential data selection biases, the number of input trajectories for odontogenesis, amelogenesis, and osteogenesis was carefully balanced. Before applying TrajDTW, we confirmed that the input trajectories retained their expected lineage-specific biological features. Canonical dentin (*Phex, Dmp1, and Dspp*), bone/mineralization (*Sp7, Runx2, Col1a1, and Alpl*), and enamel (*Klk4, Enam, and Amelx*) markers exhibited the expected tissue-specific pseudotemporal expression patterns (S9 Fig), supporting the biological validity of the trajectories used for cross-tissue comparison.

**Fig 4:**
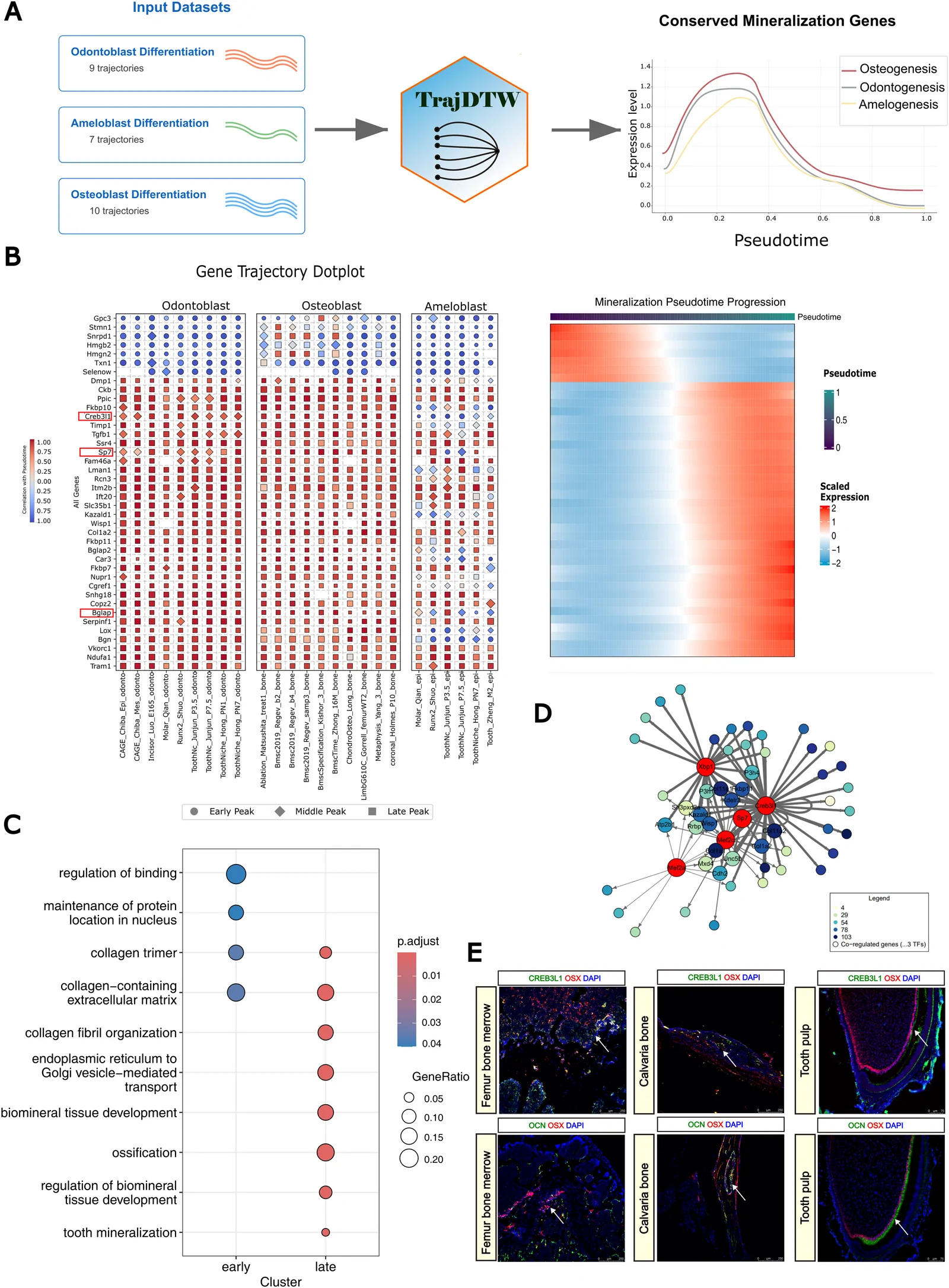
TrajDTW identifies the shared mineralization trajectory architecture across Hard tissue. (A) A schematic overview of the TrajDTW pipeline used to identify trajectory-concordant mineralization gene programs from integrated odontogenesis, amelogenesis, and osteogenesis datasets. (B) Pseudotemporal expression of trajectory-concordant gene programs. Left panel: A dot plot showing gene expression at the sample level. Dot color indicates the correlation with pseudotime, size represents expression level, and shape denotes the timing of peak expression (circle: early; diamond: middle; square: late). Right panel: A heatmap illustrating the corresponding aggregated expression patterns across the continuous pseudotime trajectory for each gene. (C) Gene Ontology (GO) enrichment analysis of gene programs with early and late expression patterns identified in (B). (D) A gene regulatory network (GRN) predicted by SCENIC. Transcription factors (TFs) are shown as red nodes, and target genes are colored according to their temporal expression pattern from (B). Node size corresponds to the number of TFs regulating the gene (indegree), and edges represent regulatory links. (E) Immunofluorescence staining of key osteogenic proteins in representative mineralizing tissues (femur bone marrow, calvarial bone, and tooth pulp). Top: Staining for the transcription factors CREB3L1 (green) and Osterix (OSX/Sp7, red). Bottom: Staining for Osteocalcin (OCN/Bglap, green) and Osterix (OSX/Sp7, red).

Following the TrajDTW analysis, the top genes with the highest trajectory concordance scores were designated as core mineralization-associated genes for further investigation (Fig 4B and S7 Table). Among the late-stage genes, *Bglap* and *Bglap2* [37] encode osteocalcin, a non-collagenous matrix protein required for proper alignment of biological apatite crystallites with collagen fibrils, whereas *Bgn* [38] encodes biglycan, an extracellular matrix proteoglycan involved in collagen fibrillogenesis, matrix organization, and osteogenic signaling (Fig 4B). Beyond these established mineralization-associated genes, TrajDTW also prioritized less-characterized candidates. For example, *Rcn3* [39], a high-concordance gene in our analysis, encodes an ER-resident calcium-binding protein implicated in collagen modification and fibrillogenesis in other matrix-producing tissues, suggesting a potential role in preparing collagen-rich extracellular matrix for mineral deposition. In contrast, a smaller subset of top-ranked genes, such as *Hmgb2* [40], showed early-stage expression patterns and may reflect upstream chromatin or progenitor-state programs associated with the transition toward mineralization commitment.

To functionally annotate these distinct expression patterns, we performed Gene Ontology (GO) enrichment analysis. This revealed that the early-pattern genes were enriched for terms such as “maintenance of protein location in nucleus,” while the late-pattern genes were significantly enriched for “biomineral tissue development,” “tooth mineralization,” and “ossification.” (Fig 4C) To identify the upstream drivers of this shared mineralization architecture, we integrated SCENIC analysis, which pinpointed key transcriptional regulators (Fig 4D). These included *Xbp1*, *Creb3l1*, *Sp7*, *Mef2c*, and *Mef2a*—all of which have previously been reported to play critical roles in regulating mineralization processes [41–45].

To experimentally validate these computational predictions, we performed Immunofluorescence Staining (IF staining) to assess the protein expression of key identified factors, including the regulators *Sp7* [46,47] and *Creb3l1* [41] and downstream gene, *Bglap* [48,49]. The results confirmed that all three proteins exhibited preferential localization in regions of active mineralization across multiple tissues, including the femur, calvaria, and tooth (Fig 4E). Quantification across three biological replicates demonstrated higher relative fluorescence intensity in mineralization regions compared with corresponding isotype IgG control sections (S12A and S12B Fig). Furthermore, these proteins exhibited significant spatial co-localization, These findings provide spatial protein-level support for their association with the shared mineralization trajectory architecture.

### Cross-species analysis supports conservation of mesenchymal/odontoblast mineralization dynamics

To establish a more comprehensive, cross-species understanding of tooth development, we constructed an extended atlas by incorporating additional contextual datasets, including human, disease-associated, and perturbation/knockout datasets. The full extended atlas comprised 391,327 cells from 59 samples across 21 projects. We utilized scANVI to integrate this expanded dataset with our core mouse (Mus musculus) reference. The analysis revealed that the majority of the collected human cells were of mesenchymal origin, with limited epithelial representation (Fig 5A). Consequently, we focused our subsequent cross-species comparisons on the mesenchymal populations.

**Fig 5:**
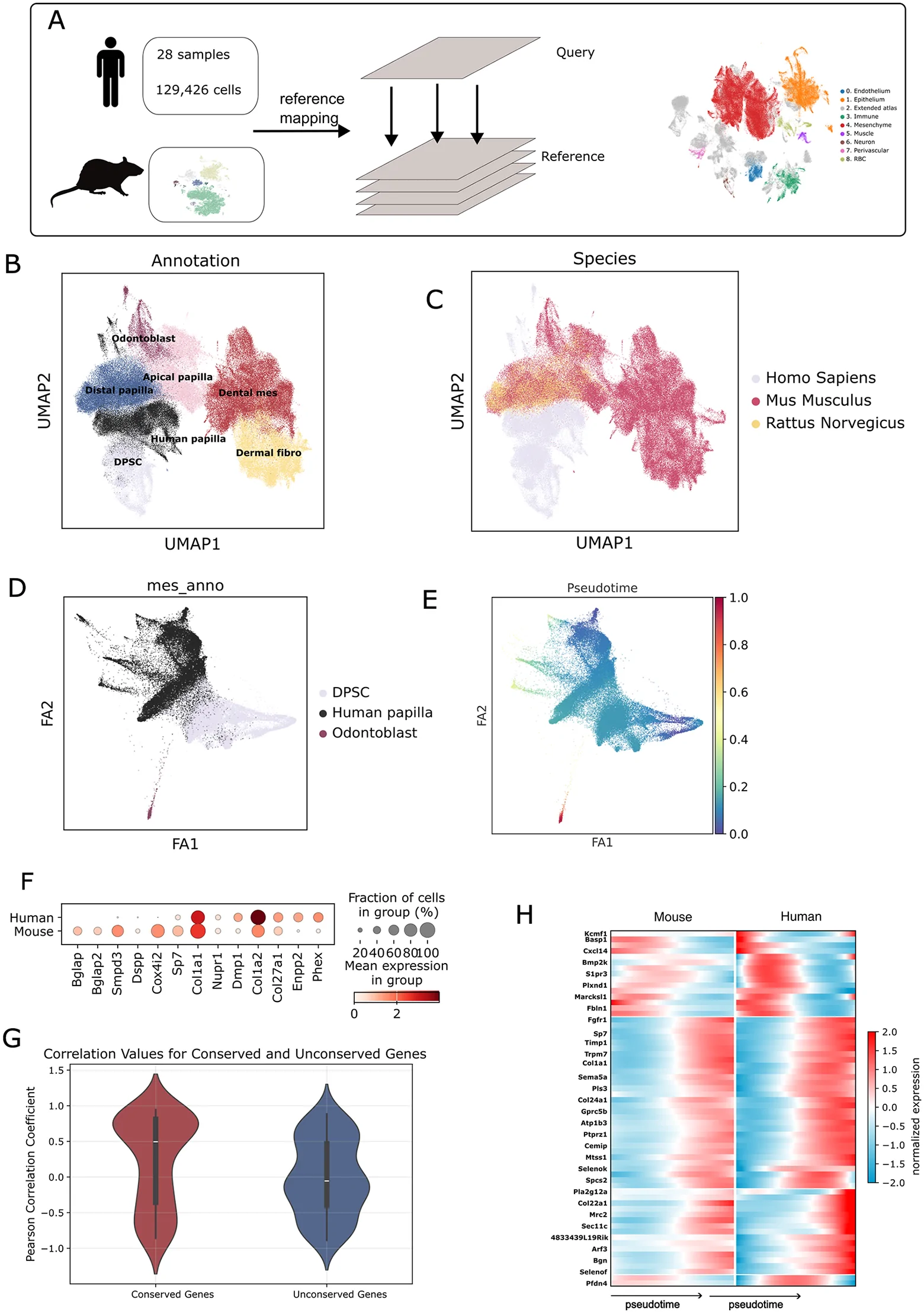
(A) Schematic of the pipeline used to generate the extended atlas. (B, C) UMAP plots of the mesenchymal clusters in the extended atlas, colored by cell annotation (B) and species (C). (D, E) Force-directed graph of the odontogenesis trajectory, with cells colored by annotation (D) and pseudotime (E). (F) Dot plot comparing the expression of key odontogenesis genes between human and mouse. (G) Pearson correlation coefficients of gene expression between human and mouse, compared for cross-species conserved versus non-conserved orthologs. (H) Heatmap comparing the pseudotemporal expression patterns of trajectory-concordant genes between human and mouse.

The analysis revealed significant differences in the mesenchymal cell states between humans and mice. While odontoblasts from both species exhibited high similarity in their cellular states and expression of canonical marker genes (e.g., *COL1A1* and *NUPR1*), the broader mesenchymal populations displayed substantial divergence in both cell-state distribution and gene expression profiles (Fig 5B, 5C and 5E). Based on established literature, we annotated these human populations as dental papilla cells and dental pulp stem/progenitor-like cells (DPSCs) (Fig 5C). Trajectory analysis further indicated that these two cell types progress along distinct differentiation paths to form mature odontoblasts in humans (Fig 5D).

A direct comparison of odontogenesis-related genes underscored these species-specific differences. For instance, key markers such as *BGLAP*, *BGLAP2*, and *SMPD3* showed significantly higher expression in mouse odontoblasts (Fig 5F). Conversely, other critical genes, including *PHEX* and *ENPP1*, were more highly expressed in their human counterparts (Fig 5F). Further functional annotation via gene ontology analysis supported these findings, revealing that human odontoblasts were enriched for terms related to “cell-substrate adhesion,” while mouse odontoblasts were enriched for “ATP biosynthetic process” (S13B Fig).

Despite these species-specific adaptations, our analysis supported conserved pseudotemporal dynamics in a subset of mesenchymal/odontoblast mineralization-associated genes across humans and mice. Among 4,000 trajectory-variable genes selected from 13,245 shared orthologs, sample-supported TrajDTW identified 53 genes that passed Wilcoxon BH-FDR and pseudotime-permutation empirical BH-FDR correction (Fig 5G). Heat-map visualization demonstrated concordant cross-species trajectories for genes including *XBP1*, an evolutionarily conserved cell-fate regulator [50], and mineralization-associated genes such as *PHEX, DMP1*, and *BMP7* (Fig 5H).

Collectively, these findings identify a shared cross-species transcriptional architecture for mesenchymal/odontoblast mineralization, revealing a fundamental and shared molecular pathway underlying vertebrate hard tissue formation.

## Discussion

In this study, we addressed a fundamental question in developmental biology by seeking to define the core molecular programs that govern hard tissue formation. By constructing an unprecedentedly large and detailed single-cell atlas of tooth development and developing a novel computational algorithm, TrajDTW, we successfully identified and validated a “shared mineralization architecture”—a core set of genes that is shared across different mineralizing tissues and conserved between mouse and human. Our work provides a unifying framework for understanding biomineralization, offers a valuable new resource and computational tool to the research community, and reveals both conserved and species-specific features of hard tissue development.

A cornerstone of our study is the creation of the most comprehensive single-cell atlas of tooth development to date. By integrating 391,327 cells from 59 samples across 21 projects, our atlas overcomes the limitations of previous, smaller-scale studies and provides unparalleled resolution. This allowed us to not only identify rare and subtle cell states, such as *C1qtnf3* + fibroblasts and *Kit*+ papilla cells, but also to meticulously dissect the entire differentiation continuum of ameloblasts into discrete pre-ameloblast, early, secretory, and mature stages. Furthermore, our atlas resolved distinct differentiation trajectories for odontoblasts originating from embryonic versus adult mesenchymal progenitors. This resource represents a significant step forward for the field, providing a foundational reference for future studies on tooth development, disease, and regeneration.

Methodologically, a key innovation of this work is the development of TrajDTW. The challenge of identifying robust biological signals from large-scale, heterogeneous single-cell datasets compiled from multiple labs is a significant bottleneck in the field. TrajDTW was specifically designed to meet this challenge by leveraging biological replicates to identify genes with the most consistent expression patterns along a given trajectory. Our multi-level validation strategy confirmed its efficacy: genes with high trajectory concordance scores were not only more tightly correlated and functionally enriched for relevant biological processes, but the algorithm’s utility was also demonstrated in an entirely different, well-characterized context—the cell cycle. By outperforming standard correlation methods on noisy synthetic data, TrajDTW establishes itself as a powerful and broadly applicable tool for any study aiming to extract concordant transcriptional dynamics from complex, large-scale single-cell experiments.

The central insight of our study is the definition of a shared mineralization trajectory architecture that unifies bone and tooth development, identified by applying TrajDTW to our integrated single-cell atlases. Our analysis revealed this program is temporally structured, progressing from early genes maintaining stemness (e.g., *Hmgb2*) to late genes driving matrix deposition (e.g., *Bglap*), with *Sp7* and *Creb3l1* emerging as conserved candidate regulatory nodes within this mineralization-associated network. We provided spatial protein-level support for this shared trajectory architecture using immunofluorescence staining, which confirmed the spatial co-localization of these factors in the active mineralization zones of both tissues, grounding our computational findings in biological reality. Crucially, our cross-species analysis demonstrated that this same mineralization axis is also partly retained across human–mouse mesenchymal/odontoblast orthologs. While we observed species-specific adaptations between human and mouse, such as divergent mesenchymal states and differential gene expression, the core set of genes within this shared mineralization trajectory architecture maintained significantly higher expression correlation. This principle of “conservation amidst divergence,” underscored by the shared dynamics of genes like *Sp7*, suggests that diverse hard tissues and species-specific adaptations are built upon an ancient and fundamental molecular scaffold for mineralization.

More broadly, recent studies illustrate how atlas-scale computation can connect descriptive molecular maps with disease modeling and translational investigation. Integrative work in oncology has shown that combining genomic, transcriptomic, epigenomic, and proteomic information across heterogeneous cohorts can help distinguish shared molecular programs from context-specific drivers and candidate vulnerabilities [51]. AI-driven multi-omics and multimodal frameworks further emphasize the potential value of linking molecular profiles with spatial, imaging, and clinical information, while also highlighting the need for domain-specific benchmarking, interpretability, uncertainty assessment, and external validation [52]. In the dental context, our atlas could serve as a reference coordinate system onto which independent disease or repair datasets are mapped. Such comparisons may help identify cell states and trajectory deviations associated with conditions such as dentinogenesis imperfecta and may prioritize candidate genes or cell populations for regenerative studies.

While our study provides a powerful resource, we acknowledge certain limitations, including potential cryptic batch effects from integrating public data and insufficient epithelial cell representation in our human dataset, which restricted a full cross-species comparison of amelogenesis. Furthermore, our findings are primarily correlational; therefore, future work involving functional perturbations, such as CRISPR-based screens or knockout models, is essential to causally dissect the roles of the novel genes identified within the mineralization axis. Nevertheless, this work opens several exciting avenues for research. The comprehensive atlas can serve as a scaffold for mapping disease states like dentinogenesis imperfecta; the TrajDTW algorithm is broadly applicable to other complex biological systems, such as neurogenesis or cancer; and the shared mineralization trajectory architecture provides a rich list of candidate genes for enhancing regenerative medicine strategies aimed at engineering functional bone and dental tissues.

In conclusion, our study provides a multi-faceted contribution by delivering a high-resolution community resource, a novel computational tool, and a unifying biological concept. We have defined a shared mineralization trajectory architecture across mouse hard tissue, thereby bridging a critical gap in developmental biology and laying the groundwork for future functional and translational research.

## Materials and methods

### Ethic statement

The study protocol was approved by the Ethics Committee for Animal Use of the Institute of Biomedical Sciences (Protocol number 69/2017). All experimental procedures were approved by Wuhan University and were performed according to laboratory animal care and use guidelines.

### Data collection

The initial core atlas collection was established using healthy tooth-related datasets. After quality control, the filtered core atlas used for primary analyses contained 261,929 cells from 31 samples across 15 projects. We further constructed an extended atlas by incorporating additional cross-species, disease-associated, and perturbation/knockout datasets. During assembly, the final merged object retained 261,901 of the 261,929 core-atlas cells; together with 129,426 query/contextual cells from 28 additional samples, this yielded 391,327 cells from 59 samples across 21 projects. Detailed data sources are provided in the S1 and S5 Table.

### Metadata collection

Comprehensive metadata were systematically collected for each sample, including age, tissue origin, tooth type, sequencing method, species, genotype, treatment conditions, tissue dissociation method, publication reference, and GEO accession number. Developmental age was categorized into five distinct stages of tooth morphogenesis: Thickening (E11-E12.5), Bud (E13-E14), Cap (E14.5-E16), Bell (E16.5-P0), and Erupted (>P0). Tissue origins were classified as mandibular or maxillary, and tooth types were categorized as incisors or molars. Histological details (e.g., dental pulp, tooth germ) were recorded when available. To account for technical variability, we documented the sequencing platform for each dataset (e.g., 10X Genomics v2, v3), as this can be a significant source of batch effects.

### Data preprocessing

Data preprocessing was performed using a previously established two-round pipeline [36]. The first round encompassed initial quality control and annotation of individual datasets. The second round involved advanced computational procedures, including droplet detection, count normalization, and selection of highly variable genes. A carefully designed batch division strategy was implemented to minimize technical artifacts while preserving biological variance, ensuring the construction of a high-quality reference atlas.

### Integration analysis

To select the integration strategy, we benchmarked commonly used methods on the pre-integration core tooth atlas, including scANVI, scVI, Harmony, Scanorama, BBKNN, Conos, Seurat RPCA, ComBat, and fastMNN. Because the methods generate embedding-, graph-, or corrected-expression outputs, the final comparison comprised 19 method/configuration entries. Batch correction was evaluated using Batch ASW, PCR batch, graph connectivity, kBET, and iLISI. Biological conservation was evaluated using NMI and ARI for cluster/label agreement, cell-type ASW, isolated-label F1 and silhouette scores, cLISI, cell-cycle conservation, and HVG conservation where applicable. Following the scIB convention, the overall score was calculated as 0.4 × batch-correction score + 0.6 × biological-conservation score (S8 Table). Mesenchymal and epithelial identity preservation was additionally evaluated separately. scANVI achieved the highest overall score while preserving both lineage compartments and was therefore used for the final atlas integration.

Following the benchmarking, we integrated the data using the scANVI model, leveraging annotations from the initial preprocessing round. The model was configured with 5,000 HVGs and 30 latent dimensions. Additional parameters were specified as follows: n_layers = 1, dispersion = “gene-batch”, encode_covariates=True, dropout_rate=0.1, gene_likelihood=”zinb”, and the model was trained for a maximum of 100 epochs.

### Cluster detection and annotation

Initial clustering was performed using the Leiden algorithm (resolution = 0.05). These clusters were then coarsely annotated (S2 Table). We then focused on the mesenchyme and epithelium superclusters for detailed annotation, as they represent the odontogenesis and amelogenesis lineages.

For fine-grained annotation, we implemented a hierarchical clustering strategy using the scHarmonization pipeline [53]. This involved generating a five-level clustering tree by applying the Leiden algorithm across a broad resolution spectrum (0.001–50) and utilizing mrtree. Sibling clusters were merged if they shared all but fewer than 10 unique marker genes (specificity > 1). The final hierarchy was visualized with ggtree. Marker genes for each cluster were identified using the van Elteren test to account for batch effects.

The top three levels of the hierarchy were manually curated, with CellHint [24] used to harmonize annotations across different studies (S3 Table). For deeper levels, cluster names were generated by concatenating the best marker gene with the parent cluster’s designation. Detailed, interactive annotations are available at https://scatlas.readthedocs.io/en/latest/annotation/index.html.

### Differential expression analysis

We performed differential expression analysis using the dreamlet package, which applies linear mixed models to sample-level pseudobulk data. Results were visualized using ComplexHeatmap, and functional enrichment analysis was conducted using ClusterProfiler.

### Trajectory inference

To delineate cellular differentiation pathways, we performed trajectory inference using a composite workflow leveraging established computational methods. The process began with the identification of trajectory origins. To achieve this, we assessed the developmental potential of each cell using three independent algorithms: CytoTRACE, CytoTRACE2, and SCENT. As all methods yielded highly concordant results, we confidently assigned progenitor populations as the starting points for the lineages. After performing dimensionality reduction using Trimap and diffusion maps, we then utilized Partition-based Graph Abstraction (PAGA) and Slingshot with these defined origins and terminal odontoblast states to infer the continuous differentiation trajectories and assign a pseudotime value to each cell. Finally, to model dynamic gene expression changes along these inferred lineages, we employed the tradeSeq [54] package to fit the expression data using generalized additive models (GAMs).

### Condition-aware comparison of embryonic and postnatal/adult odontoblast trajectories

To identify condition-dependent transcriptional dynamics, embryonic and postnatal/adult odontoblast-lineage cells were matched across five bins of a common pseudotime coordinate. Genes expressed in at least 30 cells were analyzed using a condition-aware generalized additive model implemented in tradeSeq::fitGAM, with condition-specific smoothers and five knots. The model adjusted for log10-transformed total counts, the number of detected genes, mitochondrial and ribosomal transcript fractions, C9 cell state, pseudotime bin, project, and sequencing machine. Differences between the condition-specific expression trajectories were evaluated using the global Wald test implemented in tradeSeq::conditionTest. P values were adjusted across genes using the Benjamini–Hochberg method. Genes with an adjusted P value < 0.05 were considered to exhibit significant condition-dependent trajectory dynamics. For visualization, the fitted embryo and postnatal/adult expression curves were row-scaled and grouped into eight dynamic programs using k-means clustering (k = 8, 25 starts; seed = 1234).

### TrajDTW: A tool for identifying trajectory-concordant genes across large-scale datasets

To facilitate the identification of trajectory-concordant gene expression dynamics across diverse experimental conditions and datasets, we developed TrajDTW, a novel computational framework implemented as a Python package. This framework leverages Dynamic Time Warping (DTW) to robustly compare gene expression trajectories, addressing the common challenge of identifying consistent temporal patterns in the presence of non-linear variations in differentiation or response timing. The TrajDTW workflow consists of three main stages: (1) data preprocessing and trajectory standardization, (2) calculation of a trajectory concordance score for each gene, and (3) parametric modeling of highly conserved trajectories.

### Data preprocessing and interpolation

The initial step in the TrajDTW workflow ensures that gene expression trajectories are smooth and uniformly sampled for reliable comparison. Adapting strategiesfrom the Genes2Genes [33] methodology, we employ an adaptive Gaussian kernel smoothing approach to process sparse and irregularly sampled pseudotime data. This method generates standardized trajectory matrices (dimensions: samples × time points × genes) by adjusting the kernel width based on local cell density. The weight ($W_{ij}$) of a cell j for an interpolation point i is calculated as:


$$\begin{array}{c} W_{ij}=\text{exp}\left(-\frac{{\left(t_{i}-t_{j}\right)}^{\text{2}}}{\text{2}\sigma_{i}^{\text{2}}}\right) \end{array}$$

where $t_{i}$ and $t_{j}$ are their respective pseudotime values, and $\sigma_{i}$ is the adaptive kernel width. The expression value for each gene g at interpolation point i($E_{ig}$) is then computed as a weighted average:


$$\begin{array}{c} E_{ig}=\frac{\sum_{j}W_{ij}\cdot E_{jg}}{\sum_{j}W_{ij}} \end{array}$$

For our analyses, each trajectory was standardized to 100 interpolation points. To ensure data quality, we filtered out genes and batches with poor coverage using thresholds of gene_thred = 0.1 and batch_thred = 0.3.

### Trajectory concordance scoring

With standardized trajectories, we next quantified trajectory concordance using DTW, a method adept at aligning temporal sequences despite shifts and non-linear warping. Prior to comparison, each trajectory was z-score normalized to ensure that the subsequent distance calculations were invariant to differences in expression baseline and scale. For each gene, pairwise DTW distances were calculated between all sample pairs (n) using the FastDTW algorithm (radius parameter = 3) for linear-time complexity. The DTW distance is defined as:


$$\begin{array}{c} \text{DTW}(X,Y)=\underset{\phi }{min}\sum_{i=\text{1}}^{\left|X\right|}d(x_{i},y_{\phi \left(i\right)}) \end{array}$$

where ϕ is the optimal warping path between trajectories X and Y. To enhance robustness, samples with low expression variance (below variation_threshold = 0.1) were excluded. The final trajectory concordance score for each gene was calculated as the negative logarithm of the average pairwise DTW distance, ensuring that higher scores correspond to greater trajectory concordance:


$$\begin{array}{c} \text{Concordance Score}=-{\text{log}}_{\text{1}\text{0}}\left(\frac{\text{1}}{n(n-\text{1})/\text{2}}\sum_{i=\text{1}}^{n}\sum_{j=i+\text{1}}^{n}d_{\text{DTW}}(T_{i},T_{j})\right) \end{array}$$

Where $d_{\text{DTW}}\left(T_{i},T_{j}\right)$ is the DTW distance between trajectories for samples i and j.

### Parametric modeling of high-concordance trajectories

To characterize the underlying dynamics of the highest-concordance genes, trajDTW includes a TrajectoryFitter class for parametric curve fitting. This step enables robust cross-sample comparisons and abstraction of complex expression patterns. The fitter supports multiple models (e.g., spline, polynomial, sine), with parameters optimized by minimizing the mean DTW distance between the model and the observed trajectories:


$$\begin{array}{c} \theta^{*}=\text{arg}\underset{\theta }{min}\frac{\text{1}}{n}\sum_{i=\text{1}}^{n}\text{DTW}(f\left(t;\theta \right),T_{i}) \end{array}$$

where θ represents the model parameters, f(t;θ) is the model function, and $T_{i}$ is the observed trajectory for sample i. In our study, we primarily employed cubic spline models, which offer the flexibility to capture complex biological patterns while maintaining robustness against noise and batch effects.

### Synthetic trajectory benchmark

Each benchmark unit comprised paired condition-A and condition-B trajectories generated from one of four smooth latent families: monotonic increase, early peak, late peak, or transient pulse. Within each stratum, 12 positive pairs represented four trajectory families across three latent variants. Positive condition-B trajectories preserved the corresponding latent program under either a global pseudotime shift or a monotone nonlinear warp. For each positive pair, ten matched negative pairs retained the structured condition-A trajectory but replaced condition B with pseudotime-independent random values exactly matched to the positive condition-B trajectory in finite-sample mean and standard deviation.

Each stratum therefore contained 12 positive and 120 negative pairs. Each condition comprised four replicates with 80 cells per replicate. Gaussian measurement noise was added with standard deviation σ times the standard deviation of the latent condition-A trajectory. The noise benchmark used σ = 0.25, 3.0, 4.0, and 6.0. The shift and nonlinear-warp benchmarks used σ = 3.0; global displacement ranged from Δ = 0.00 to 0.30, and the tested nonlinear maps comprised identity, power, S-curve, piecewise, and multi-segment transformations evaluated at matched observed warp areas.

TrajDTW was compared with aligned Pearson correlation, CV similarity, and Genes2Genes. Within each simulation seed, the shift and warp strengths shared the same latent trajectories, pseudotime samples, measurement-noise draws, and matched negative realizations. Five independent seeds were evaluated. Because the positive-to-negative ratio was 1:10, discrimination was summarized primarily using average precision, together with full precision–recall curves and descriptive maximum-F1 values. Mean average precision and 95% t intervals were reported across seeds for the shift and warp benchmarks. AUROC was retained as a secondary metric.

### Cross-species analysis

For the cross-species analysis, we used the additional query/contextual component of the extended atlas, comprising 129,426 cells from 28 samples. To harmonize these datasets, we employed scArches [55] to project the query samples onto a previously trained reference model, thereby mapping them into a shared latent space. Cell type annotations were then transferred from the reference atlas to the query cells using a k-nearest neighbors (KNN) classifier. For all subsequent trajectory analyses, we isolated the mesenchymal cell populations in silico.

Differentiation trajectories of the mesenchymal lineage were reconstructed by calculating Diffusion Pseudotime (DPT). The pseudotime ordering was computed based on the cell-to-cell transition probabilities within the force-directed graph embedding. To identify orthologous genes with conserved pseudotemporal dynamics, we performed a cross-species comparison between human and mouse datasets. Specifically, we selected the 4,000 most trajectory-variable genes from 13,245 shared mouse–human orthologs and calculated TrajDTW distances across 50 mouse–human sample pairs per gene. Genes with distances consistently below 0.90 were identified using a one-sided Wilcoxon test followed by BH correction. Human pseudotime labels were then permuted 1,000 times to calculate empirical P values and BH-FDR across all 4,000 genes. The resulting 53 conserved genes were visualized using row-scaled fitted trajectories.

### Mice

Female C57BL/6 mice were obtained from GemPharmatech (Nanjing, China). The mice were sacrificed by anesthesia overdose at 8 weeks before harvesting. Calvaria, tooth and femur were fixed in 4% paraformaldehyde at 4°C for 24 h and then decalcified with 10% EDTA (pH 7.4) for 14–21 days, and then embedded in paraffin.

### Immunofluorescence staining

Immunofluorescence staining was performed on formalin-fixed, paraffin-embedded (FFPE) tissue specimens. The specimens were sectioned at 4–6 μm, deparaffinized, and rehydrated. A multiplex immunofluorescence protocol using Tyramide Signal Amplification (TSA) was employed to detect Creb3l1 (Boster, PB0513; 1:200), Bglap (ABclonal, A20800; 1:200), and Sp7 (Abcam, ab209484; 1:200). The staining was conducted in sequential cycles. For the first cycle, antigen retrieval was performed via microwave heating in a commercial retrieval solution (Servicebio, G1201), followed by blocking with 5% normal donkey serum. Sections were then incubated with the first primary antibody overnight at 4°C. Following washes in phosphate-buffered saline (PBS), sections were incubated with an HRP-conjugated secondary antibody (1:200) for 1 h at 37°C, and the signal was developed using a TSA-fluorophore kit (Histova Biotechnology, DFT4C100) for 60 seconds. To prepare for the next antigen, the bound primary and secondary antibodies were stripped by microwave heating in the same retrieval solution for 20 min at 95°C. This cycle of primary antibody incubation, secondary antibody/TSA detection, and antibody stripping was repeated for each subsequent target. Upon completion of all staining cycles, nuclei were counterstained with DAPI, and slides were coverslipped with mounting medium. Image acquisition was performed on a Leica Thunder Imager 3D Assay system, and images were subsequently processed and analyzed using Leica Application Suite X software (v3.5.7, Leica).

### Quantitative immunofluorescence analysis

Isotype IgG negative-control sections were processed in parallel under identical acquisition and image-processing settings. Images from three independent biological replicates per tissue were analyzed in ImageJ. Fluorescence channels and DAPI were separated, and regions of interest were defined using DAPI-positive tissue areas. Regions of interest (ROIs) corresponding to the mineralization regions and anatomically matched regions in the negative-control sections were manually delineated according to DAPI-positive tissue morphology. Background-subtracted mean fluorescence intensity (MFI) was measured for CREB3L1, OSX, OCN and DAPI. Relative fluorescence intensity was calculated as the ratio of background-corrected target fluorescence intensity to DAPI fluorescence intensity to normalize for differences in tissue area and nuclear density among sections. Values are presented as mean ± SD. Group differences were assessed by one-way ANOVA followed by Tukey’s post-hoc test, with the significance thresholds defined in the figure legend. Statistical comparisons were performed separately for each marker.

## Supporting information

S1 Fig Dataset composition and metadata structure of the tooth atlas. (A) Metadata collection framework, including sample-level biological and technical covariates. (B) Sample-level cell counts across the initial collection, filtered core atlas, and extended atlas. Each bar represents one sample; the y-axis shows log2-transformed cell number, colors indicate source projects, and annotations indicate species, atlas subset, and filtering status.
(TIF)

S2 Fig (A) Preprocess pipeline of tooth atlas construction. (TIF)

S3 Fig Benchmarking and hyperparameter optimization for single-cell data integration. (A) Nineteen method/configuration combinations were evaluated for batch correction and biological conservation using scIB metrics. The comparison summarizes performance across global, mesenchymal, and epithelial evaluations and supports scANVI as the best-balanced integration approach. (B) Benchmarking results for scANVI hyperparameters, specifically the number of highly variable genes (HVGs) and latent dimensions (n_latent) (C-H) Boxplots showing the results of fine-tuning individual scANVI parameters on performance metrics.
(TIF)

S4 Fig Composition and annotation of the integrated tooth atlas. (A) Histogram displaying the percentage of each cell type within the integrated atlas.(B) Frequency of cell type occurrences across the different projects included in the atlas.(C) Dot plot of canonical marker genes for each cell type. Dot size represents the proportion of cells expressing the gene, and color indicates the average expression level. (D) Harmonization of cell type annotations using CellHint. Cell annotations from individual studies were harmonized into a unified set of labels.
(TIF)

S5 Fig Age-associated changes in dental mesenchyme. (A) UMAP visualization of mesenchymal cells, colored by developmental stage and cell type annotation. (B, C) Heatmaps displaying age-upregulated (B) and age-downregulated (C) genes in the dental follicle. (D) Volcano plot showing differentially expressed genes (DEGs) in the dental follicle with age. (E) Gene Ontology (GO) analysis of age-upregulated and age-downregulated genes from the dental follicle. (F, G) Heatmaps displaying age-upregulated (F) and age-downregulated (G) genes in the apical papilla. (H) Volcano plot showing DEGs in the apical papilla with age. (I) GO analysis of age-upregulated and age-downregulated genes from the apical papilla.
(TIF)

S6 Fig Lineage trajectories in tooth development. (A) PAGA plot illustrating potential differentiation trajectories from dental mesenchymal cells and Apical Papilla towards an odontoblast fate. (B,C) Diffusion map visualization of the mesenchymal and epithelial cell populations. (D) UMAP visualization of mesenchymal cells colored by expression of lineage-specific marker genes: Msx2 (Adult) and Prrx1 (Embryo). (E) UMAP feature plots showing the cluster-specific expression of key amelogenesis-related genes.
(TIF)

S7 Fig Stemness and pathway analysis in tooth development. (A, B) Stemness assessment of mesenchymal cells using CytoTRACE2 (A) and SCENT (B). In the CytoTRACE2 plot (A), red indicates a less differentiated state.(C, D) Stemness assessment of epithelial cells using CytoTRACE2 (C) and SCENT (D). Similarly, red in (C) indicates a less differentiated state.(E, F) Heatmap displaying signaling pathway activity scores during the odontogenesis (E) and amelogenesis (F) processes.
(TIFF)

S8 Fig Functional annotation of dynamic gene programs. (A,B) Gene ontology enrichment analysis of gene clusters in Fig 2G (A) and 2H (B).
(TIF)

S9 Fig Canonical mineralization-marker trajectories across odontoblast, osteoblast, and ameloblast samples. Dot plots show dentin markers (*Phex, Dmp1, Dspp*), bone/mineralization markers (*Sp7, Runx2, Col1a1, Alpl*), and enamel markers (*Klk4, Enam, Amelx*) across trajectory samples. Dot size denotes normalized trajectory expression, color denotes correlation with pseudotime, and shape denotes the timing of peak expression; crosses indicate genes absent from the source tensor.
(TIF)

S10 Fig Synthetic benchmarks of TrajDTW for trajectory correspondence. (A) Examples of global temporal shifts and monotone nonlinear pseudotime warps. (B) Synthetic trajectories under increasing Gaussian measurement noise. (C) Matched-negative and temporally shifted trajectory examples. (D) Average-precision comparison of TrajDTW, aligned Pearson correlation, CV similarity, and Genes2Genes across Gaussian-noise levels. (E) Mean average precision across increasing global pseudotime displacement. (F) Mean average precision across matched-area power, S-curve, piecewise, and multi-segment nonlinear warps. Error bars in E–F denote 95% t-based confidence intervals across five independent simulation seeds; the dotted horizontal line denotes the positive-class prevalence of 0.091. (G) Median scoring wall time across increasing gene and replicate-pair workloads, measured over five repeats.
(TIF)

S11 Fig Benchmarking of TrajDTW. (A) Scatter plot correlating the TrajDTW conservation score with the log fold change from differential expression analysis. Each point represents a gene. Genes highlighted in red boxes were selected as representative conserved and unconserved genes. (B) Gene Ontology (GO) enrichment analysis of genes categorized by their conservation and differential expression in (A). (C) Schematic of the data processing pipeline for the cell cycle dataset. (D) GO enrichment analysis of conserved versus unconserved genes identified by TrajDTW within the cell cycle dataset. (E) Violin plots comparing Pearson correlation coefficients of gene expression patterns between individual datasets and the overall consensus pattern. Conserved genes show a significantly higher correlation than unconserved genes.
(TIF)

S12 Fig Immunofluorescence imaging and quantitative analysis of mineralization markers in skeletal and dental tissues. (A) Representative immunofluorescence images showing CREB3L1/OSX/DAPI (upper panels) and OCN/OSX/DAPI (lower panels) staining in mouse femoral bone marrow, calvaria, and tooth tissues. Isotype IgG control sections were processed in parallel under identical staining and imaging conditions. Representative images of non-mineralized and mineralized regions are shown. Green represents CREB3L1 or OCN, red represents OSX, and blue represents DAPI. (B) Quantification of relative fluorescence intensity (RFI = target MFI/DAPI MFI) for CREB3L1, OSX, and OCN in calvarial, femoral, and dental tissues. Blue represents IgG control/non-mineralized regions and red represents mineralized regions. Data are presented as mean ± SD from three independent biological replicates. Statistical significance was determined using one-way ANOVA followed by Tukey’s post-hoc test. *P < 0.05, **P < 0.01, ***P < 0.001, ****P < 0.0001.
(TIF)

S13 Fig Composition and cross-species analysis of the extended atlas. (A) The cellular composition of the extended atlas, showing the relative proportion of each cell type. (B) Gene Ontology (GO) enrichment analysis of genes differentially expressed between human and mouse odontoblasts.
(TIF)

S1 Table Metadata for public single-cell datasets of tooth development. (XLSX)

S2 Table Marker genes used for broad cell type annotation. (CSV)

S3 Table Literature-curated marker genes for annotating single-cell clusters in the tooth atlas. (CSV)

S4 Table Differentially expressed genes between embryonic and adult dental follicle and apical papilla. (XLSX)

S5 Table Metadata for public single-cell datasets used in the cell cycle analysis. (XLSX)

S6 Table List of canonical cell cycle genes curated from the literature. (XLSX)

S7 Table TrajDTW-derived trajectory concordance scores for genes across mouse odontoblast, ameloblast, and osteoblast trajectories. (CSV)

S8 Table Metrics table of scIB pipeline. (TSV)
